# Dentin dysplasia type I—A dental disease with genetic heterogeneity

**DOI:** 10.1111/odi.12861

**Published:** 2018-04-10

**Authors:** D Chen, X Li, F Lu, Y Wang, F Xiong, Q Li

**Affiliations:** ^1^ Department of Stomatology The First Affiliated Hospital of Zhengzhou University Zhengzhou China; ^2^ Department of Medical Genetics School of Basic Medical Sciences Southern Medical University Guangzhou China

**Keywords:** dentin dysplasia, pathogenic genes, *SMOC2*, *SSUH*2, *VPS4B*

## Abstract

Hereditary dentin disorders include dentinogenesis imperfecta (DGI) and dentin dysplasia (DD), which are autosomal dominant diseases characterized by altered dentin structure such as abnormality in dentin mineralization and the absence of root dentin. Shields classified DGI into three subgroups and DD into two subtypes. Although they are all hereditary dentin diseases, they do not share the same causative genes. To date, the pathogenic genes of DGI type I, which is considered a clinical manifestation of syndrome osteogenesis imperfecta, include *COL1A1* and *COL1A2*. Mutations of the *DSPP* gene, which encodes the dentin sialophosphoprotein, a major non‐collagenous protein, are responsible for three isolated dentinal diseases: DGI‐II, DGI‐III, and DD‐II. However, DD‐I appears to be special in that researchers have found three pathogenicity genes―*VPS4B*,*SSUH2*, and *SMOC2―*in three affected families from different countries. It is believed that DD‐I is a genetically heterogeneous disease and is distinguished from other types of dentin disorders. This review summarizes the DD‐I literature in the context of clinical appearances, radiographic characteristics, and functions of its pathogenic genes and aims to serve clinicians in further understanding and diagnosing this disease.

## 1 INTRODUCTION

Heritable dentin defects are rare diseases affecting both deciduous and permanent teeth (Nieminen et al., 2011), which present with abnormally mineralized dentin. Based on clinical manifestations and imaging features, these diseases were classified into dentinogenesis imperfecta (three subtypes: DGI‐I, DGI‐II, and DGI‐III) and dentin dysplasia (two subtypes: DD‐I and DD‐II) (Shields, Bixler, & El‐Kafrawy, 1973). However, a large number of clinical studies have reported that teeth in DGI‐I, DGI‐II, DGI‐III, and DD‐II patients share analogous characteristics of amber translucent crowns accompanied by significant attrition, which extends even to the alveolar crest, with short and thin roots with obliterated pulp (Rafeek, Paryag, & Al‐Bayaty, 2013), while DD‐I teeth always appear normal shape and crown color, but are accompanied by sharp or absent root (Khandelwal & Likhyani, 2014). Subsequently, molecular biology and genetics studies have shown that *DSPP* is the pathogenic gene of DGI‐II, DGI‐III, and DD‐II, but not DD‐I. On account of the clinical manifestations and the mutant gene, investigators believe that the Shields classification is no longer applicable and have proposed a new classification: dentinogenesis imperfecta (DI, DSPP‐related disease) and radicular dentin dysplasia (Shields DD‐I) (de La Dure‐Molla, Philippe Fournier, & Berdal, 2015). DGI‐I is not involved in this new classification because it is not an independent dental disease but an oral manifestation of osteogenesis imperfecta. This new classification is consistent with the Mendelian Inheritance in Man (MIM) database, including DGI‐II (MIM 125490), DGI‐III (MIM 125500), DD‐I (MIM 125400), and DD‐II (MIM 125420). This new systematic classification system appears to be more scientific and reasonable.

Despite the fact that DD‐I is a special dentin genetic disease, there is no literature describing the disease in detail in terms of definition, classification, clinical features, etiology, diagnosis, differential diagnosis, or treatment. DD‐I is a rare disease with an autosomal dominant pattern of inheritance (Shields et al., 1973) that affects either the primary or both the primary and the secondary dentitions with an incidence of 1/100,000 (Kalk, Batenburg, & Vissink, 1998). The condition was first described as “rootless teeth” by Ballschmiede (Chamberlain & Hayward, 1983) in 1920; however, it was Rushton who named the condition “dental dysplasia” in 1939 (Malik, Gupta, Wadhwan, & Suhasini, 2015). Patients with DD‐I always present with either mobile teeth or pain associated with numerous periapical radiolucencies in non‐carious teeth (Khandelwal & Likhyani, 2014). In this review, we summarize the clinical appearance, histological performance, functions of the pathogenic genes, diagnostic criteria, differential diagnosis, and treatment of DD‐I to help clinicians gain a better understanding of this disease and to develop reasonable treatment plans.

## 2 CLINICAL AND HISTOLOGICAL DESCRIPTION

DD‐I teeth exhibit extreme mobility and generally premature exfoliation as a result of conical or absent roots (Ye et al., 2015), even though they are apparently normal in morphology and color. Other frequent symptoms and complaints, such as delayed dental eruption pattern, opaque incisal margins (Kalk et al., 1998; Scola & Watts, 1987), spontaneous exfoliation, and discomfort caused by severe tooth mobility, especially after meals (Ozer, Karasu, Aras, Tokman, & Ersoy, 2004), have also been reported. Based on its radiographic characteristics, DD‐I has been classified into four subtypes. Type 1a has no pulp chamber or root formation and always exhibits periapical radiolucency. Type 1b exhibits short roots a few millimeters in length, with single small crescent or herringbone shape pulp and periradicular radiolucencies. Type 1c has shortened inside roots, in which a central island of dentine is surrounded by horizontal or vertical crescent‐shape pulpal remnants and variable periapical radiolucencies. In addition to periapical radiolucencies, type 1d exhibits roots of normal length with a visible pulp chamber and large pulp stones located in the coronal portion of the canal (Carroll, Duncan, & Perkins, 1991; Shields, 1983).

Histologically, the affected teeth manifest normal coronal enamel with a thin layer of subjacent normal dentin (Sauk, Lyon, Trowbridge, & Witkop, 1972). Deeper layers of dentin exhibit an atypical tubular pattern with an amorphous, atubular area, and irregular arrangement. More centrally arranged dysplastic dentin masses may suggest multiple pulp calcifications (Rocha, Nelson‐Filho, Silva, Assed, & Queiroz, 2011; Toomarian, Mashhadiabbas, Mirkarimi, & Mehrdad, 2010). The dentinoenamel junction is either smooth or scalloped, and the scalloped structure is usually larger than the dentinoenamel junction of normal teeth. Ultrastructural study of the affected teeth reveals teardrop‐shape lacunae near the cervical enamel, fewer dental tubules, and irregular collagen fibers (Pintor et al., 2015; Ye et al., 2015).

## 3 IDENTIFICATION OF PATHOGENIC GENES IN DD

**Figure 1:**
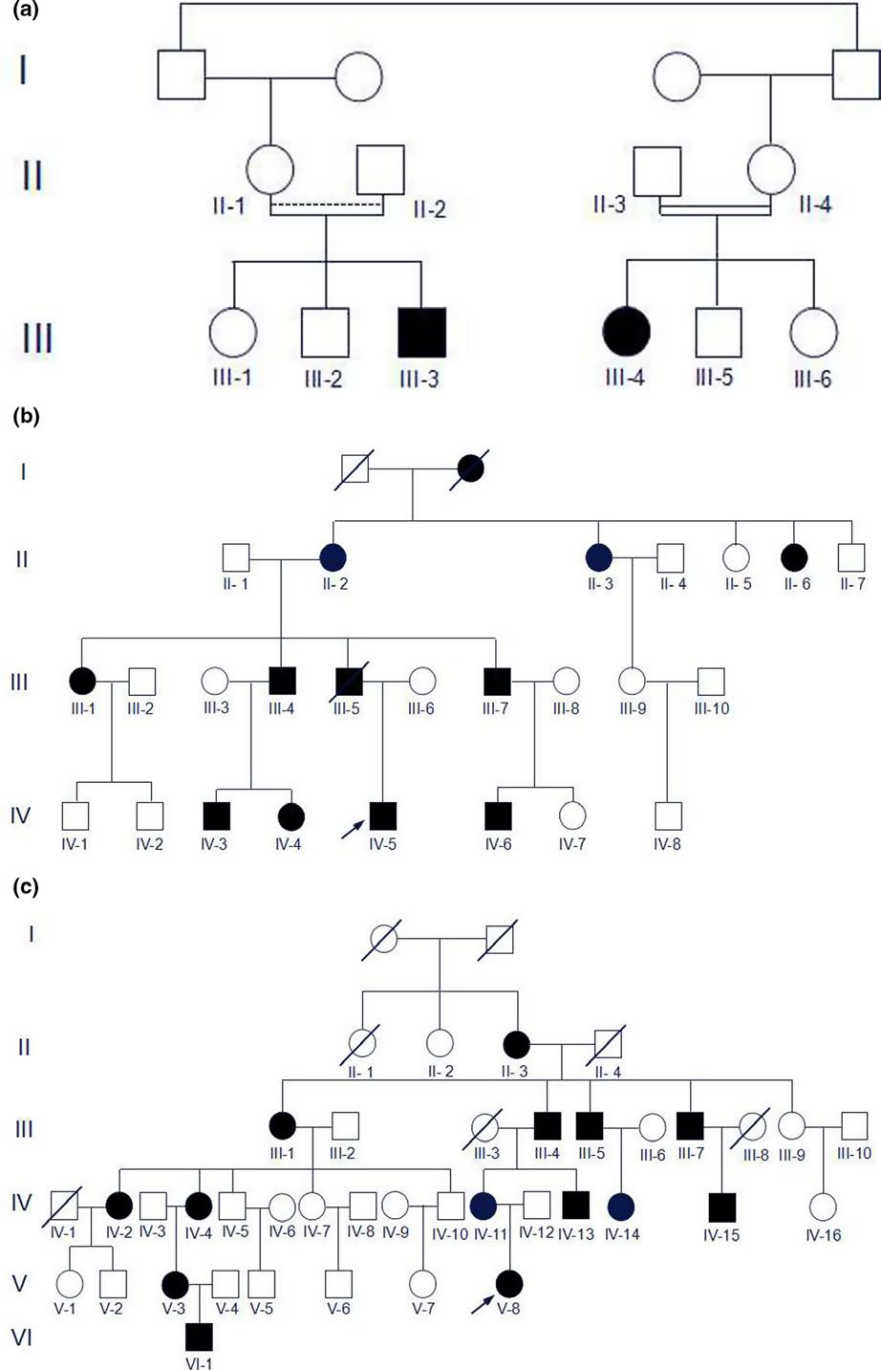
The pedigree maps of the three affected families. (a) Family with SMOC2 mutations. (b) Family with VPS4B mutations. (c) Family with SSUH2 mutations. The pedigree A is from the literature “Homozygosity Mapping and Candidate Prioritization Identify Mutations, Missed by Whole‐Exome Sequencing, in SMOC2, Causing Major Dental Developmental Defects” which is published in “The American Journal of Human Genetics” [Colour figure can be viewed at wileyonlinelibrary.com]

Investigators widely speculated about the pathogenesis of DD in earlier studies because the pathogenic gene involved in the disease was unknown. (Logan, Becks, Silverman, and Pindborg (1962) believed that variation of the dentin papillae caused abnormal tooth development, and calcification in the dentin papilla caused final occlusion of the pulpal space. Sauk et al. (1972) suggested the invagination of the root sheath occurred too soon during root development and, in a series of futile attempts to correct itself, resulted in ectopic dentin formation. Witkop (1975) proposed that internal cells of the developing dental organ would be displaced and proliferate in the dental papilla, producing ectopic dentin formation. On the other hand, Wesley, Wysoki, Mintz, and Jackson (1976) hypothesized that an ectopic interaction between odontoblasts and ameloblasts would occur, causing differentiation and/or abnormal function of the odontoblasts. Recently, three mutant genes have been detected in three affected pedigrees with different genetic modes. (Table 1, Figure 1).

**Table 1:** Pathogenic genes of DD‐I

Gene name and location	Mutant position	cDNA	Mutant type	Reference
*SMOC2* (NM_022138, 6q27)	Intron 1	c.84 + 1G>T	Splice mutation	Bloch‐Zupan et al. (2011)
*VPS4B* (NM_004869.3, 18q21.33)	Intron 7	IVS7 + 46C>G	Splice mutation	Yang et al. (2016)
*SSUH2* (NM_015931.2, 3p26.1)	Exon 2	c.353C>A	Missense mutation	Xiong et al. (2017)

**Figure 2:**
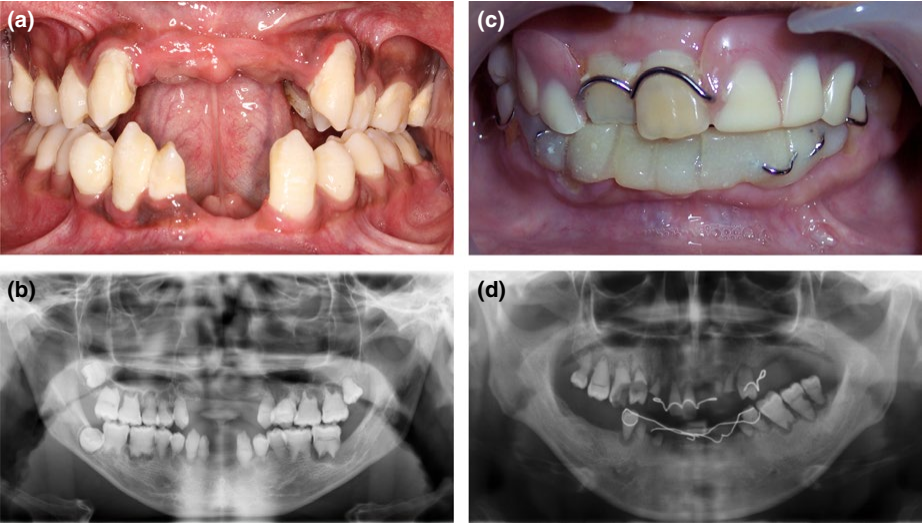
Clinical features of the patients. (a) Intraoral photo shows taurodontic teeth. (b) Panoramic radiograph revealed rootless teeth and periapical cysts. (c) A patient with normal crowns. (d) Panoramic radiograph revealed deformed root with unexplained apices

**Figure 3:**
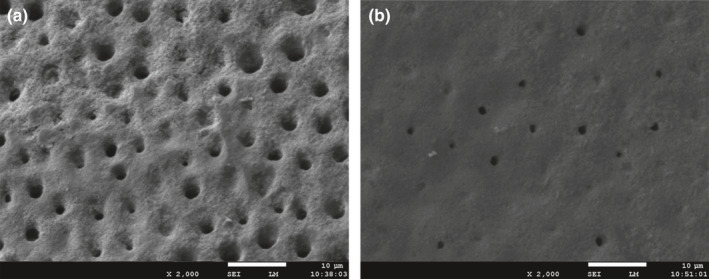
SEM examination of the teeth specimens. (a) Regularly arranged dentin tubules were found in normal teeth (2000 × ). (b) Sparsely scattered dentin tubules with reduced diameter were found in DD‐I teeth (2000 × )

In 2011, Bloch‐Zupan reported a case of homozygous mutant *SMOC2* in a family in which patients exhibited short roots similar to those in DD‐I teeth (Bloch‐Zupan et al., 2011). It is intriguing that the mode of inheritance in their pedigree was consistent with autosomal recessive rather than dominant. However, other scholars have also reported an autosomal recessive DD‐I family with no mutations in the *SMOC2* gene (Cherkaoui Jaouad et al., 2013). The study has been stalled until our research team found two new DD‐I families. The patients of the two families manifested the typical clinical symptoms of normal or taurodontic teeth and deformed root with unexplained apices (Figure 2a–d). The results of scanning electron microscopy revealed a significantly reduced number of dentin tubules with smaller diameters (Figure 3a,b). In addition, we have found two mutant genes: *VPS4B* (Yang et al., 2016) in a family from northern China; and *SSUH2* in a family from southern China (Xiong et al., 2017). Different mutations can result in the same disease which is highly suggestive evidence that the disease has genetic heterogeneity.

## 4 FUNCTIONAL AND PATHOGENIC MECHANISM OF THESE GENES

### 4.1 *SMOC2*

*SMOC2* encompasses 14 exons encoding the secreted protein acidic and rich in cysteine (SPARC)‐related modular calcium‐binding protein‐2 (SMOC2) and has been mapped to chromosome 6q27. SMOC2, and is a member of the SPARC family, which is highly expressed during embryogenesis and wound healing (Vannahme, Gosling, Paulsson, Maurer, & Hartmann, 2003). As a matricellular protein, SMOC2 can promote matrix assembly and stimulate endothelial cell proliferation and migration as well as angiogenic activity (Bornstein & Sage, 2002). The encoded protein may serve as a target for controlling angiogenesis in tumor growth and myocardial ischemia. SMOC2 appeared to have a developmental function similar to that in the root of human teeth in a mouse model (Kim et al., 2016).

However, the specific function of SMOC2 in tooth development remains unknown. Two families with the *SMOC2* mutation have been reported, and patients in both families usually exhibit extreme microdontia, oligodontia, dental shape anomalies, and short roots (Alfawaz et al., 2013). Using the well‐established morpholino knockdown technique, researchers found that the *smoc2* gene affected the development of teeth by regulating the expression of related genes, including *dlx2b, bmp2a, and pitx2* in zebrafish (Bloch‐Zupan et al., 2011).

### 4.2 *VPS4B*

Vacuolar protein sorting 4B (VPS4B) is encoded by the *VPS4B* gene, which is located on chromosome 18q21.33, and is a member of the AAA protein family (ATPases associated with diverse cellular activities). It is the homolog of the yeast VPS4 protein. VPS4B is a component of the endosomal sorting complexes required for transport (ESCRT) machinery (Gan & Gould, 2011), which has been shown to play an important role in the formation of multivesicular bodies (MVBs), virus budding (Watanabe et al., 2007), abscission of cytokinesis (Morita et al., 2010), and degradation of various membrane receptors (Jiang et al., 2015; Liu, Lv, et al., 2013). In addition, it also serves as a putative adaptor domain for the ESCRT‐III complex, which mediates the final abscission step of cytokinesis in mammals and archaea (Stuchell‐Brereton et al., 2007). The *VPS4B* gene is widely expressed in the human body, including pulp tissue (Bueno et al., 2011), and has been found to be a tumor suppressor gene that can regulate tumor progression. It is reported that VPS4B serves as a tumor suppressor in breast cancer via promoting the degradation of epidermal growth factor receptor (Lin et al., 2012). In addition, VPS4B can regulate the progression of non‐small‐cell lung cancer (Liu, Lv, et al., 2013), play a role in Parkinson's disease (Hasegawa et al., 2011), participate in neuronal apoptosis after cerebral ischemia in a middle cerebral artery occlusion model (Cui et al., 2014), and also facilitate intestinal epithelial cell apoptosis in Crohn's disease via the p38 MAPK signaling pathway (Zhang et al., 2015). Clearly, VPS4B is a highly multifunctional protein, although its expression and potential functions in tooth development remain unclear.

**Figure 4:**
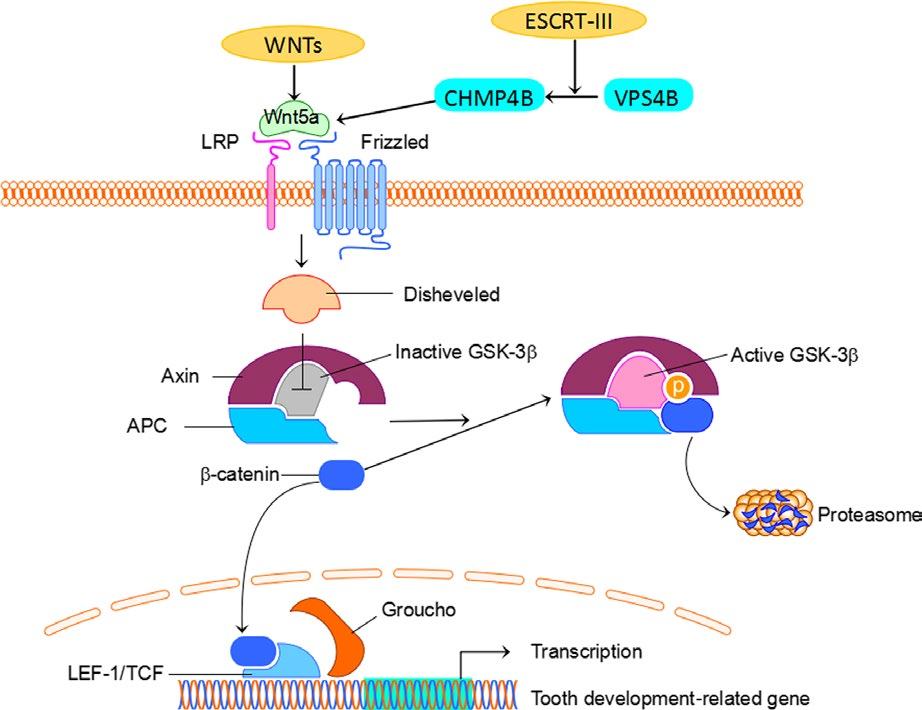
VPS4B affects tooth development‐related gene expression through Wnt/β‐catenin pathway. VPS4B regulation of CHMP4B stimulates Wnt5a, which associates with the receptor Frizzled and coreceptor LRP to trigger the Wnt signaling pathway. Wnt5a stimulates disheveled to inhibit GSK3β as well as prevent the phosphorylation of β‐catenin. Non‐phosphorylated β‐catenin induces the expression of related factors of odontoblast differentiation to further regulate root development

According to a study involving a family from northern China, the mutant gene was identified as *VPS4B* and was located on chromosome 18. Research has shown that the expression levels of VPS4B messenger RNA (mRNA) were significantly reduced in patients compared with normal controls. The overexpression of *VPS4B* is accompanied by a significant increase in the levels of *CHMP4B*,* Wnt5a*, and β*‐catenin* mRNA, while the knockdown of *VPS4B* inhibits the expression of *CHMP4B*,* Wnta5a*, and β*‐catenin* mRNA (Yang et al., 2016). Charged multivesicular body protein 4b (CHMP4B), a subunit of ESCRT‐III, in association with Wnt5a, activates the β‐catenin‐independent/Wnt pathway involved in cytokinesis (Fumoto, Kikuchi, Gon, & Kikuchi, 2012; McCullough, Fisher, Whitby, Sundquist, & Hill, 2008). The Wnt signaling pathway plays a pivotal role in the formation of root dentin and the cementum (Chen, Lan, Baek, Gao, & Jiang, 2009; Liu, Han, Wang, & Feng, 2013; Zhang et al., 2013). The *VPS4B* gene combined with CHMP4B can promote Wnt5a expression, thereby regulating the Wnt signaling pathway and controlling root formation (Figure 4). Experiments have shown that the knockdown of the *vps4b* gene in zebrafish can result in reduced tooth numbers and shorter teeth, mimicking the DD‐I phenotype. Simultaneously, the zebrafish mutant phenotype could be partially corrected by wild‐type human *VPS4B* mRNA; however, overexpression of wild‐type and mutant *VPS4B* mRNA had no significant effect on zebrafish tooth development (Yang et al., 2016). Therefore, it is speculated that the phenotype in zebrafish is caused by haploinsufficiency; however, the clinical pathogenesis in humans needs further investigation.

### 4.3 *SSUH2*

*SSUH2*, which is also known as *SSU‐2, fls485*, and *C3orf32*, maps to chromosome 3p26.1. It was first identified in a complementary DNA library prepared from fetal liver mRNA, which includes at least three open reading frames and is presumed to encode various translation products with different possible functions (Reinartz et al., 2010). However, no data concerning the functional relevance of *SSUH2* are currently available. *SSUH2* has been hypothesized to be a candidate tumor suppressor gene due to its close location to the uveal melanoma susceptibility locus UVM2 at 3p25 (Tschentscher et al., 2003). Additionally, it is also believed to be a possible chaperone with thiol‐disulfide oxidoreductase activity involved in the pathogenesis of celiac disease (Reinartz et al., 2010).

To identify essential transcription factors in dental development, specific primers with different junctions at the three mRNA spliceosomes were designed for RT‐PCR amplification. Only the transcript NM_015931.2 including a 1,803 base pair mRNA is highly expressed in human dental pulp stem cells and underjaw tissues (Xiong et al., 2017). This transcript corresponds to a putative chaperone protein (approximately 39 kDa), which is widely distributed (Reinartz et al., 2010). In addition, subcellular localization analysis shows that the SSUH2 protein is a nuclear protein and, as such, researchers hypothesize that it might be a transcription factor involved in transcriptional regulation or protein–protein interactions in tooth formation. Animal experiments have further clarified the pathogenic mechanism of this protein. In zebrafish, the incidence of agomphosis after morpholino knockdown of *ssuh2* was higher than that in the control and could be partially rescued by the introduction of the wild‐type gene; meanwhile, the mutant *ssuh2* gene downregulated the expression of three other reported tooth markers (*dlx2b, bmp2a, and pitx2*) (Xiong et al., 2017). Additionally, the expression of *Bmp2* and *Dlx2* was downregulated, while that of *Dspp, Dmp1, Pax9*, and *Runx2* was dramatically upregulated in *Ssuh2* ^+/−^ and *Ssuh2* ^−/−^ mice (Xiong et al., 2017). These findings indicate that *SSUH2* may participate in a dental development‐related signaling pathway involving several genes.

## 5 DIAGNOSIS AND DIFFERENTIAL DIAGNOSIS

In general, diagnosis is based on history, clinical manifestations, and radiographic features (Khandelwal & Likhyani, 2014). DD‐I teeth appear normal in morphology and color but show extreme mobility as a result of conical or absent roots. Radiographic features include sharp conical roots, pulp obliteration with crescent‐shape pulpal remnants parallel to the cementoenamel junction, or total obliteration. Numerous periapical radiolucencies in non‐carious teeth are diagnostic factor for this disorder.

DD‐I should be differentiated from DD‐II and DGI, which are characterized by amber translucent crown, and short and thin roots with obliterated pulp. In addition, some systemic disorders that exhibit features similar to dental DD‐I need to be differentiated. Disease involving skeletal anomalies, which is inherited as an autosomal dominant trait, is described by Morris and Augsburger (1977). These patients manifest not only the features of DD‐I, but also dense sclerotic bone and skeletal anomalies of the wrists and hand bones. Vitamin D‐dependent rickets type I (VDDR‐I) and vitamin D‐resistant rickets (VDRR) are characterized by a metabolic disturbance that causes defective calcification of mineralized structures. Patients with VDDR‐I exhibit not only abnormal teeth with short roots, large pulp chambers, and a widened predentin layer, but also short stature, skeletal abnormalities, genu valgum, rachitic rosary, open fontanels, pathologic fractures, muscle weakness, and convulsions (Zambrano et al., 2003). Features of VDRR include bowing of the legs, impaired growth, short stature, and dentin defects such as large pulp chambers, short roots, and abscessed non‐carious primary or permanent teeth (Souza et al., 2013). Chemotherapy or irradiation to the jaws during the period of root development leads to delayed root development and can exhibit radiographic features of DD‐I (Fayle, Duggal, & Williams, 1992). Familial tumoral calcinosis is a rare familial disorder characterized by masses of calcification in periarticular soft tissues. Teeth in patients manifest as short bulbous roots, obliterated pulp, and periapical radiolucencies in non‐carious teeth (Erbudak, Akkaya, Özbek, Hamdi Çelik, & Tatar, 2016).

## 6 TREATMENT AND PROGNOSIS

Treatment aims to remove infection, preserve existing teeth, enhance occlusion, and restore esthetics (Singh, Gupta, Yuwanati, & Mhaske, 2013). Due to the genetic heterogeneity of the disease, the treatment varies according to the severity of the problem and presenting complaints. Strategies should include conventional endodontic therapy, periapical curettage, and/or a preventive regimen (Khandelwal & Likhyani, 2014). However, extraction has also been suggested as a treatment option for teeth with pulp necrosis and periapical abscess (Malik et al., 2015). In the primary dentition, an overdenture can help maintain the occlusal vertical dimension. Treatment involving dental implants may be considered at approximately 18 years of age. Additionally, regular oral examination and routine conservative treatment, such as caries prevention, are essential (Ravanshad & Khayat, 2006). The outcome in patients with DD‐I always depends on his/her age and the severity of the disease. Early exfoliation of the teeth may lead to maxillomandibular atrophy in DD‐I. If the diagnosis can be made early and the treatment is appropriate, satisfactory esthetics and function can be achieved.

## 7 CONCLUSION

Dentinal genetic diseases have been described clinically for several years and have low incidence, similar to other hereditary pathologies. Elucidation of the genetic basis of dentin disorders should permit a better understanding of disease etiology, enabling improved classification, diagnosis, and treatment of this disease. By summarizing existing knowledge regarding the pathogenic genes of these diseases, de La Dure‐Molla proposed a new classification: DI (DSPP‐related disease) and radicular dentin dysplasia (Shield DD‐I). To date, three pathogenic genes have been found in different families, indicating that DD‐I has genetic heterogeneity. This feature can adequately explain the phenomenon that different patterns of inheritance exist in the affected families. Although DD‐I is a rare disease, clarifying its pathogenesis is necessary because dentin mineralization and root formation play important roles during tooth development. Although a series of pathogenic genes have been identified, detailed pathogenic mechanisms remain unclear. Further exploration of genetic function and signaling pathways is needed to acquire a comprehensive understanding of these diseases.

## CONFLICT OF INTERESTS

None to declare.

## AUTHOR CONTRIBUTIONS

Xiaocong Li composed the initial draft of the manuscript and figures. Dong Chen extended the text and edited the final manuscript. Fangli Lu and Yingying Wang edited the figures. Fu Xiong and Qiang Li reviewed and revised the manuscript to improve grammar and clarity.
